# Acute Effects of Different Blood Flow Restriction Protocols on Bar Velocity During the Squat Exercise

**DOI:** 10.3389/fphys.2021.652896

**Published:** 2021-06-21

**Authors:** Michal Wilk, Robert Trybulski, Michal Krzysztofik, Grzegorz Wojdala, Yuri Campos, Adam Zajac, Ewelina Lulińska, Petr Stastny

**Affiliations:** ^1^Institute of Sport Sciences, Jerzy Kukuczka Academy of Physical Education, Katowice, Poland; ^2^Department of Medical Sciences, The Wojciech Korfanty School of Economics, Katowice, Poland; ^3^Provita Zory Medical Center, Zory, Poland; ^4^Postgraduate Program, Faculty of Physical Education and Sports, Federal University of Juiz de Fora, Juiz de Fora, Brazil; ^5^Study Group and Research in Neuromuscular Responses, Federal University of Lavras, Lavras, Brazil; ^6^Faculty of Physical Education, Gdansk University of Physical Education and Sport, Gdańsk, Poland; ^7^Department of Sport Games, Faculty of Physical Education and Sport, Charles University, Prague, Czechia

**Keywords:** occlusion, resistance exercise, cuff, peak velocity, performance

## Abstract

The main goal of the present study was to evaluate the effects of different blood flow restriction (BFR) protocols (continuous and intermittent) on peak bar velocity (PV) and mean bar velocity (MV) during the squat exercise at progressive loads, from 40 to 90% 1RM. Eleven healthy men (age = 23.4 ± 3.1 years; body mass = 88.5 ± 12.1 kg; squat 1RM = 183.2 ± 30.7 kg; resistance training experience, 5.7 ± 3.6 years) performed experimental sessions once a week for 3 weeks in random and counterbalanced order: without BFR (NO-BFR), with intermittent BFR (I-BFR), and with continuous BFR (C-BFR). During the experimental session, the participants performed six sets of the barbell squat exercise with loads from 40 to 90% 1RM. In each set, they performed two repetitions. During the C-BFR session, the cuffs were maintained throughout the training session. During the I-BFR, the cuffs were used only during the exercise and released for each rest interval. The BFR pressure was set to ∼80% arterial occlusion pressure (AOP). Analyses of variance showed a statistically significant interaction for MV (*p* < 0.02; η^2^ = 0.18). However, the *post hoc* analysis did not show significant differences between particular conditions for particular loads. There was no significant condition × load interaction for PV (*p* = 0.16; η^2^ = 0.13). Furthermore, there were no main effects for conditions in MV (*p* = 0.38; η^2^ = 0.09) as well as in PV (*p* = 0.94; η^2^ = 0.01). The results indicate that the different BFR protocols used during lower body resistance exercises did not reduce peak bar velocity and mean bar velocity during the squat exercise performed with various loads.

## Introduction

Blood flow restriction (BFR) is a training method that reduces arterial and venous blood flow during effort (Takano et al., 2005; Loenneke and Pujol, 2009; Scott et al., 2015). The pressure of external compression used for BFR should be adjusted based on the individual value of arterial pressure where the value of 100% arterial occlusion pressure (AOP) is the point where blood flow is completely cut off. There are different possibilities to use BFR in the training process, such as continuous BFR used during several sets of exercise without releasing the pressure during rest intervals, intermittent BFR used during several sets of an exercise while releasing pressure during the rest interval, and preconditioning BFR used only before exercise (Incognito et al., 2016; Marocolo et al., 2016, 2017, 2018, 2019; Wilk et al., 2018, 2020d, 2020e). The use of BFR in resistance training increases acute physiological responses such as metabolic stress (Pearson and Hussain, 2015; Teixeira et al., 2018), cell swelling (Loenneke et al., 2012b), and endocrine responses (Takano et al., 2005; Shimizu et al., 2016). It is likely that BFR similarly to ischemia preconditioning enhances ATP production by glycolytic and phosphogenic pathways (Janier et al., 1994; Mendez-Villanueva et al., 2012) as well as peak contractile force (Andreas et al., 2011). Since the level of explosive performance depends on these substrates and metabolic mechanisms (Robergs et al., 1991), the use of BFR may be an effective tool for developing muscular power. However, the potential beneficial effect of BFR will depend on several training variables (type of exercise, number of repetitions and sets performed, load used) as well as the scheme, duration, and the level of pressure applied during BFR. In addition to the physiological effects, mechanical factors related to BFR can potentially increase acute physical performance (Rawska et al., 2019; Wilk et al., 2020d, e).

Although much attention has been focused on resistance exercise under BFR, only three previous studies have examined its acute effect on power output or bar velocity (Gepfert et al., 2020; Wilk et al., 2020a, e), and only one examined its effects in lower limbs (Gepfert et al., 2020). The results of the study by Gepfert et al. (2020) showed that BFR significantly increases power output and bar velocity during the squat exercise, but only when used at extremely high pressure equal to 150% AOP. However, such improvement in power performance was not observed under BFR with a pressure of 100% AOP. On the other hand, the same experimental procedure with a slightly lower BFR pressure (90% AOP), yet used for the upper limbs, significantly increased the power output and bar velocity during the bench press exercise at 70% 1RM (Wilk et al., 2020e). Therefore, the increase in compression pressure of 10% AOP for the lower limbs, as in the study of Gepfert et al. (2020), compared to that of Wilk et al. (2020e) (upper limbs), was not sufficient to enhance power output and bar velocity. In this regard, it seems that greater pressure is required in the lower limbs to completely close blood flow (Crenshaw et al., 1988; Loenneke et al., 2012b; McEwen et al., 2019), which also applies to the improvement of explosive performance. Furthermore, according to a study by Gepfert et al. (2020), the positive effect of BFR on power output and bar velocity may depend not only on cuff width and circumference of the limbs but also on the length of the limb. The lower limb requires a higher BFR pressure and wider cuff to induce an acute increase in performance similar to that observed for the upper limbs (Gepfert et al., 2020).

Wilk et al. (2020a) showed that continuous and intermittent BFR used during the bench press exercise significantly increased peak bar velocity for loads from 20 to 50% 1RM but not for loads above 60% 1RM. Therefore, the acute impact of BFR on power performance is related to the external load used during resistance exercise. Furthermore, the results of the study by Wilk et al. (2020a) did not record a negative effect of BFR on the mean velocity under any load (20–90% 1RM) compared to the control conditions. Thus, the use of BFR in a wide range of external loads does not degrade the efficiency of power performance during the bench press exercise. However, it is still unknown whether these acute gains during resistance exercise with BFR and low load can also be observed in case of lower limbs. The barbell squat exercise (SQ) is one of the most demanding and effective exercises for lower limb hypertrophy, strength, and power development (Myer et al., 2014; Lee and Lee, 2018). As mentioned before, greater external loads for lower limbs, due to their larger circumference, require a higher pressure or wider cuffs to induce a comparable performance enhancement observed in upper limbs. Considering the above, it would be interesting to examine whether the use of continuous and intermittent BFR with wider cuffs affect bar velocity during the squat exercise and whether the acute effects of BFR are modified by the external load used.

BFR as a training tool can be used as an additional factor to help athletes and coaches in programming varied resistance training protocols, which could help athletes break through plateaus and prevent training monotony. Previous studies have confirmed the beneficial acute effect of BFR on upper-limb explosive performance; however, the effects are dependent on the external load used. It seems reasonable to determine whether similar benefits of BFR will occur during lower-limb resistance exercise and whether these effects will be related to the external load used. Therefore, the main goal of the present study was to examine the acute effects of different exercise protocols of BFR (continuous and intermittent) on bar velocity during the squat exercise with progressive loads. It was hypothesized that both protocols of BFR enhance bar velocity during the squat exercise.

## Materials and Methods

### Study Design

The randomized crossover experiments were performed in the Strength and Power Laboratory at the Academy of Physical Education in Katowice, Poland, in accordance with the procedure used in the study of Wilk et al. (2020a). Each participant took part in three different experimental protocols, 1 week apart: without BFR (NO-BFR), with intermittent BRF (I-BFR), and with continuous BFR (C-BFR). In C-BFR, the cuffs were maintained throughout the experimental session. In I-BFR, the cuffs were applied only during the exercise and were released during each rest interval. The BFR pressure was set to ∼80% AOP. During each experimental session, the participants performed six sets of the squat exercise, for two repetitions in each set, at loads progressing from 40 to 90% 1RM. There were 3-min rest intervals between sets. The repetition was performed with a controlled tempo of movement, consisting of a 2-s eccentric phase and maximum speed concentric phase of the movement.

### Subjects

Eleven healthy men volunteered for the study (age = 23.4 ± 3.1 years; body mass = 88.5 ± 12.1 kg; SQ 1RM = 183.2 ± 30.7 kg). The following inclusion criteria were used in the study: a 1RM in the squat exercise over 150% of body weight and that the participant was free from physical activity contraindications and free from musculoskeletal injuries. Participants were informed about the main purpose of the study, potential risks and benefits, and gave their written consent to participate in the experiment. In addition, participants were able to withdraw from the study at any time. The Bioethics Committee for Scientific Research, at the Academy of Physical Education in Katowice, Poland, approved the experimental protocol (02/2019).

### Familiarization Experimental Sessions

Three weeks before the main experiment in order to minimize possible learning effects, the participants performed two familiarization sessions. During the familiarization session, the participants performed three sets of the squat exercise under BFR, at a load of 40, 60, and 80% of their estimated 1RM. In each set, two repetitions were performed.

### 1RM Squat Strength Test

One week before the main experiment, the 1RM squat test was performed according to the recommendations proposed by Gepfert et al. (2020). On this day, participants arrived at the laboratory at the same time of the day as in the upcoming experimental sessions (in the morning, between 9:00 and 11:00). Upon arrival, the subject cycled on an ergometer for 5 min at an exercise intensity that induced a heart rate of ∼130 bpm. Next, the subject performed the squat exercise, lunges, crunches, and torso hyperextensions with their own body weight and self-selected number of repetitions. After the general warm-up the participants performed 10 repetitions at 20 kg, 6 repetitions at 60%, 4 repetitions at 70%, and 3 repetitions at 80% of their estimated 1RM. The first testing load was set to ∼90% 1RM. Next the load was increased by 2.5–10 kg for each subsequent set, until the participant was unable to perform a concentric phase of movement. In each 1RM attempt, the participant performed one repetition with a controlled tempo of movement, consisting of a 2-s eccentric phase and maximum speed concentric phase of the movement (Wilk et al., 2020b). Between each 1RM attempt, there was a 5-min rest interval.

### Experimental Sessions

During the experimental sessions, the participants performed the squat exercise under three different conditions: without BFR (NO-BFR), with intermittent BFR (I-BFR), and with continuous BFR (C-BFR). During each experimental session, the participants performed the squat exercise for a total of six sets at progressive load: 40, 50, 60, 70, 80, and 90% with a 3-min rest interval between sets. The cuff pressure for the squat exercise was set to ∼80% of full arterial occlusion. The movement tempo was controlled and consistent with that used in the 1RM test. For the evaluation of bar velocity (peak and mean), we used the linear position transducer system (Tendo Power Analyzer, Tendo Sport Machines, Trencin, Slovakia) (Garnacho-Castaño et al., 2015). During each repetition, peak bar velocity (m/s) and mean bar velocity (m/s) were registered. Mean bar velocity (MV) was obtained as the mean of the two repetitions, while peak bar velocity (PV) was obtained from the best repetition in each set. During the squat exercise, technical criteria were applied in accordance with Martínez-Cava et al. (2019) and according to the rules of the International Powerlifting Federation (Wilk et al., 2020c).

### Procedure of Blood Flow Restriction

The BFR cuffs “Smart Cuffs” (10 cm; Smart Tools Plus LLC, Strongsville, OH, United States) were placed in the most proximal part of both legs. The cuff pressure for the squat exercise was set to ∼80% of full arterial occlusion (amounting 215 ± 22 mm Hg). For I-BFR condition, the BFR was used only during the exercise and released during each rest interval. For I-BFR condition, the cuff was applied immediately before the squat set and released upon completion of the two repetitions [total duration of I-BFR, ∼30 s (∼5 s of effort for each set × 6 sets)]. For C-BFR condition, the cuffs were applied 1 min before the start of the first experimental set and were held until the experimental session, also during the rest intervals [total duration of C-BFR, ∼17 min (∼5 s of effort for each set, six sets, 3-min rest intervals)].

### Statistical Analysis

To verify the normality, homogeneity, and sphericity of the sample data variances, we used the Shapiro–Wilk test. Differences between the conditions (NO-BFR vs. I-BFR vs. C-BFR) were examined using repeated measures two-way analyses of variance (3 conditions × 6 loads). Effect sizes (ES) for main effects and interactions were calculated by partial eta squared (*η*^2^). Partial eta-squared values were classified as large > 0.137, moderate from 0.137 to 0.06, and small from 0.059 to 0.01. The Tukey’s *post hoc* test was used when a main effect or interaction occurred. For pairwise comparisons, the Cohen’s d test was used (large *d* > 0.8, moderate d between 0.8 and 0.5, small d between 0.49 and 0.20, trivial *d* < 0.2; Cohen, 1988). Percent changes with 95% confidence intervals (95% CIs) were also calculated. All statistical analyses were performed using Statistica 9.1.

## Results

The two-way ANOVA demonstrated statistically significant interaction for MV (conditions × load; *p* < 0.02; η^2^ = 0.18; Table 1). However, the *post hoc* analysis did not show significant differences between conditions for particular loads.

**TABLE 1:** Mean bar velocity during the bench press exercise for six different loads.

Condition	40% 1RM (95% CI)	50% 1RM (95% CI)	60% 1RM (95% CI)	70% 1RM (95% CI)	80% 1RM (95% CI)	90% 1RM (95% CI)
**Peak bar velocity (m/s)**						
NO-BFR	1.04 ± 0.11 (0.96–1.11)	0.94 ± 0.12 (0.86–1.03)	0.83 ± 0.10 (0.76–0.90)	0.73 ± 0.08 (0.68–0.79)	0.65 ± 0.08 (0.59–0.70)	0.53 ± 0.06 (0.49–0.57)
I-BFR	1.01 ± 0.11 (0.94–1.09)	0.92 ± 0.09 (0.86–0.98)	0.83 ± 0.09 (0.77–0.89)	0.75 ± 0.06 (0.71–0.80)	0.66 ± 0.08 (0.60–0.71)	0.57 ± 0.06 (0.53–0.61)
C-BFR	1.00 ± 0.11 (0.93–1.08)	0.93 ± 0.10 (0.87–0.99)	0.83 ± 0.08 (0.78–0.89)	0.73 ± 0.10 (0.66–0.79)	0.61 ± 0.08 (0.55–0.67)	0.53 ± 0.07 (0.48–0.58)
**Effect size**						
NO-BFR vs. I-BFR	0.27	0.19	0.0	0.28	0.13	0.67
NO-BFR vs. C-BFR	0.36	0.09	0.0	0.0	0.50	0.0
I-BFR vs. C-BFR	0.09	0.11	0.0	0.24	0.63	0.61

*Results are expressed as mean ± SD (95% confidence intervals). 1 RM, 1 repetition maximum; NO-BRF, no blood flow restriction (control); I-BFR, intermittent blood flow restriction; C-BFR, continuous blood flow restriction.*

The two-way ANOVA did not show significant condition × load interaction for PV (*p* = 0.16; η^2^ = 0.13; Table 2) and did not show significant main effect for conditions for MV (*p* = 0.38; η^2^ = 0.09; 0.77 vs. 0.79 vs. 0.77 m/s for NO-BFR, I-BFR, and C-BFR, respectively) and for PV (*p* = 0.94; η^2^ = 0.01; 1.45 vs. 1.46; 1.45 m/s for NO-BFR, I-BFR, and C-BFR, respectively). The moderate effect size (ES) in MV was found between NO-BFR vs. C-BFR and between I-BFR vs. C-BFR at a load of 80% 1RM as well as between NO-BFR vs. I-BFR and between I-BFR vs. C-BFR at load 90% 1RM.

**TABLE 2:** Peak bar velocity during the bench press exercise for six different loads.

Condition	40% 1RM (95% CI)	50% 1RM (95% CI)	60% 1RM (95% CI)	70% 1RM (95% CI)	80% 1RM (95% CI)	90% 1RM (95% CI)
**Peak bar velocity (m/s)**						
NO-BFR	1.72 ± 0.24 (1.56–1.88)	1.64 ± 0.27 (1.46–1.83)	1.48 ± 0.19 (1.35–1.61)	1.38 ± 0.21 (1.24–1.52)	1.27 ± 0.18 (1.15–1.39)	1.19 ± 0.15 (1.09–1.29)
I-BFR	1.73 ± 0.25 (1.56–1.90)	1.60 ± 0.21 (1.46–1.74)	1.52 ± 0.18 (1.40–1.64)	1.41 ± 0.13 (1.32–1.50)	1.29 ± 0.13 (1.21–1.38)	1.19 ± 0.10 (1.12–1.25)
C-BFR	1.72 ± 0.24 (1.55–1.88)	1.67 ± 0.19 (1.53–1.80)	1.52 ± 0.15 (1.42–1.62)	1.39 ± 0.15 (1.28–1.49)	1.26 ± 0.16 (1.15–1.36)	1.16 ± 0.14 (1.07–1.26)
**Effect size**						
NO-BFR vs. I-BFR	0.04	0.17	0.22	0.17	0.13	0.0
NO-BFR vs. C-BFR	0.0	0.13	0.23	0.05	0.06	0.21
I-BFR vs. C-BFR	0.04	0.35	0.0	0.14	0.21	0.25

*Results are expressed as mean ± SD (95% confidence intervals). 1 RM, 1 repetition maximum; NO-BRF, no blood flow restriction (control); I-BFR, intermittent blood flow restriction; C-BFR, continuous blood flow restriction.*

## Discussion

The main finding of the study was that both intermittent and continuous BFR does not change mean and peak bar velocity for all tested loads, which is contrary to the initial hypothesis. However, even if the BFR conditions do not increase squat performance, it should be emphasized that BFR does not have any negative effects on power performance at any load. Therefore, the results of the presented study allow the use of BFR within a wide range of external loads, without reduction in bar velocity in short-term resistance exercises.

Currently, only one previous study has analyzed the acute effect of BFR on bar velocity and power output during the squat exercise with wide cuffs (Gepfert et al., 2020), identical to that used in the present study (10 cm). The study by Gepfert et al. (2020) showed that bar velocity and power output were significantly higher for the squat at 70% 1RM under BFR but only when extremely high cuff pressure was used (150% AOP). When a lower cuff pressure (100% AOP) was used, there was no difference in bar velocity between BFR and control conditions (Gepfert et al., 2020), which is consistent with the presented study. However, this comparison was made for only one value of the external load (70% 1RM), while the presented study assessed a wide range of loads (40–90% 1RM). Furthermore, what is especially important for the C-BFR condition is the fact that heavier loads were performed after longer occlusion (12–17 min) compared to lighter loads (only 1 min BFR for 40% 1RM and 4–5 min for 50% 1RM). Thus, a direct comparison of the presented results to studies in which a constant external load was used is not equivalent.

The experimental procedures consisting of a wide range of loads (similar order of loads as in our study) were used in the study by Wilk et al. (2020a), yet in an upper-limb exercise. The study by Wilk et al. (2020a) demonstrated that intermittent as well as continuous BFR increased peak bar velocity during the bench press exercise at loads of 20, 30, 40, and 50% 1RM but not for loads above 60% 1RM, which is contrary to the presented results, where no differences in bar velocity were observed (at any loads) between BFR and control conditions. Conflicting results of the presented research and the study of Wilk et al. (2020a) may be related to a different area of cuff placement (lower vs. upper limbs). The lower limb requires a higher pressure or wider cuff to induce a similar acute improvement in performance than the upper limbs due to the circumference ratio and the ratio of limb length to cuff width (Crenshaw et al., 1988; Loenneke et al., 2012b; McEwen et al., 2019; Wilk et al., 2020a). However, even though a wider cuff was used in the present study, compared to the experiment of Wilk et al. (2020a) (10 vs. 4 cm), this width difference was not sufficient to induce changes in bar velocity during the squat exercise. It seems that to induce a significant effect on bar velocity in lower limb exercises, not only should a wider cuff be used, but at that same time, cuff pressure should be increased. This has been confirmed by the research results of Wilk et al. (2020e) and Gepfert et al. (2020). Wilk et al. (2020e) reported an increase in power performance during the bench press exercise at 70% 1RM when he used BFR with 90% AOP. Despite using the same device, “Smart Cuffs,” with the same width of 10 cm, in the lower limbs, as in the study by Gepfert et al. (2020), the results did not show such an increase in performance in the squat exercise with BFR under the pressure of 100% AOP. Therefore, the 10% AOP increase in cuff pressure for the lower limbs (Gepfert et al., 2020) compared to the upper limbs (Wilk et al., 2020e) was insufficient to enhance power output and bar velocity during the squat exercise. However, increasing the AOP to 150% during BFR was sufficient to induce performance enhancement during the squat (Gepfert et al., 2020), but a pressure > 100% AOP due to complete blood flow cut off should be used only in short-term intermittent BFR (Wilk et al., 2018). Therefore, in order to increase squat performance, extremely high pressure should be used, but for health reasons, this is only possible with an intermittent exercise protocol of BFR.

Although the presented study did not register increases in bar velocity for both BFR conditions (intermittent and continuous), the results showed no negative effect on performance under any load compared to control conditions. The lack of differences, especially between the C-BFR and control conditions, is surprising, as there was a large difference in the total duration of the BFR. The C-BFR lasted approximately 17 min, but it did not decrease the squat power performance. Furthermore, despite the large difference between the C-BFR and I-BFR in the duration of time under BFR (approximately 17 min vs. 30 s, respectively, for C-BFR and I-BFR), the results did not show differences in bar velocity at any loads. However, it should be assumed that even if there were no differences in kinematic variables between conditions, there were significant differences in physiological, hormonal, and metabolite postexercise responses. For example, Torma et al. (2019) found that BFR used during rest periods of squat exercise at 70% 1RM in 7 sets with 10 repetitions appears to enhance the gene expression of angiogenesis, mitochondrial biogenesis, muscle repair, and hypertrophy in healthy young men. These findings could be of great importance to athletes involved in various sports. It seems that BFR may contribute to enhancing beneficial postexercise physiological responses without compromising physical performance. Moreover, it seems that the C-BFR increases physiological and metabolic stress, which consequently may cause increased fatigue and potentially decrease exercise performance (Wernbom et al., 2009; Loenneke et al., 2012a; Pearson and Hussain, 2015; Neto et al., 2018; Teixeira et al., 2018; Wilk et al., 2018; Okita et al., 2019). However, despite the increased fatigue in the C-BFR and I-BFR compared to the control, there was no negative effect on bar velocity during the squat exercise at any external load. This may be due to the short time under tension in each sets (∼5 s) and the fact that such a short effort mainly uses phosphocreatine (Bird et al., 2005), which is quickly and extensively restored during rest intervals between sets (Bogdanis et al., 1996; Dawson et al., 2007). Therefore, during short efforts, BFR, as well as schemes of BFR application (I-BFR and C-BFR), do not enhance power performance during the squat exercise.

Although BFR during resistance exercise increases metabolic stress and endocrine responses (Takano et al., 2005; Pearson and Hussain, 2015; Shimizu et al., 2016; Teixeira et al., 2018), the presented studies did not show a decrease in bar velocity during BFR conditions. It can be assumed that BFR, through the mechanical work generated by the cuff, could compensate the potential decrease in squat performance resulting from progressive fatigue. Previous research suggested that the mechanical work produced by the cuff may be an additional factor that may affect the effectiveness of resistance exercise under BFR, especially when using high external compression (Rawska et al., 2019; Gepfert et al., 2020; Wilk et al., 2020e). The cuff is passive, yet during movement, especially during the eccentric phase, the tension in the cuff material can create additional elastic energy, which can increase the efficiency of the concentric phase of movement (Rawska et al., 2019; Gepfert et al., 2020; Wilk et al., 2020e). Moreover, the placebo and/or nocebo effect is a methodological factor that may have a significant impact on acute effects of BFR. Marocolo et al. (2016) showed a similar increase in performance during resistance exercise for the BFR condition with cuff pressure of 220 mm Hg and for the placebo condition with cuff pressure equal to 20 mm Hg. Therefore, the reduction in the potential decrease in squat performance for BFR conditions resulting from arising fatigue can be related to mechanical work generated by the cuff as well as the placebo effect.

It should be noticed that the potential increase in postexercise physiological responses under BFR may cause an increase in postexercise muscle damage or rhabdomyolysis (Wernbom et al., 2019, 2020). Possible muscle damage following training under BFR could slow down the recovery process and may affect the quality of subsequent training sessions. Unfortunately, in the present study, we did not assess the level of postexercise biochemical markers, gene expression, hormonal responses, or the level of muscle damage that may have occurred in the days following the exercise sessions, which consists a significant limitation of the study. Another limitation is that the order effect (load) was not taken into account, which limits the possibility of comparing the BFR effect between the used loads. Therefore, these results cannot be translated into other types of exercises as well as to other training procedures. This study assessed the acute impact of BFR on explosive performance in a strictly defined training protocol (six sets of the squat exercise, for two repetitions in each set, at loads progressing from 40 to 90% 1RM). The different training protocol could cause different responses. Therefore, further research is needed, especially with regard to between-load comparisons, using a research design in which the order of the loads used is randomized (to account for the order effect).

### Practical Implications

The present study showed that resistance exercises in various BFR application procedures did not increase the concentric phase of the bar movement during the squat exercise. Despite the lack of performance improvement under BFR conditions, it should be noted that the C-BFR condition may induce additional physiological responses, such as metabolic stress, gene expression, enhancement of intramuscular signaling, endocrine responses, and increased recruitment of fast twitch muscle fibers. Therefore, maintaining peak and mean concentric velocity during a multiset squat exercise under BFR, while possibly increases physiological responses (not evaluated in the present study), may be a significant factor determining the level of postexercise adaptive changes.

However, it should be noted that exercise under BFR can also cause a number of side effects, especially if used too often. The disadvantages relate to the long-term use of BFR in one muscle area and mainly relate to the weakening of the musculature at the site of immediate cuff placement (Kacin and Strazar, 2011; Ellefsen et al., 2015). Therefore, when BFR is used, the training protocols should be carefully planned with gradual progression of loads over time, to ensure that protective adaptations (i.e., a repeated bout effect) can occur in order to minimize the risk of excessive muscle stress and damage (Clark and Manini, 2017; Wernbom et al., 2019). Furthermore, it must be stated that muscle BFR does not have to be used in every set or every exercise of a particular training session. It is a common training practice to use different schemes of BFR, in different exercises, as well as with different BFR pressure in successive training sessions. BFR training protocols should vary during particular microcycles to prevent monotony and overtraining.

## Conclusion

The results of this study indicate that the BFR used during lower body resistance exercises does not impair concentric peak and mean bar velocity during the squat exercise with loads ranging from 40 to 90% 1RM. Furthermore, the applied BFR protocol did not impact power performance during the SQ exercise at any load. Although the presented study did not register increases in bar velocity for both BFR conditions (intermittent and continuous), the results showed no negative effects on performance under any load for BFR compared to NO-BFR condition.

## Data Availability Statement

The raw data supporting the conclusions of this article will be made available by the authors, without undue reservation.

## Ethics Statement

The study protocol was approved by the Bioethics Committee for Scientific Research, at the Academy of Physical Education in Katowice, Poland (02/2019). The patients/participants provided their written informed consent to participate in this study.

## Author Contributions

MW and RT: study conception and design. MW, GW, and EL: acquisition of data. MW, MK, and PS: analysis and interpretation of data. MW, RT and YC: drafting of manuscript. MW, MK, RT and AZ: critical revision. All authors contributed to the article and approved the submitted version.

## Conflict of Interest

The authors declare that the research was conducted in the absence of any commercial or financial relationships that could be construed as a potential conflict of interest.
