# Supplementary material for: Acute Effects of Different Blood Flow Restriction Protocols on Bar Velocity During the Squat Exercise
Source: Front Physiol. 2021 Jun 21;12:652896. doi: 10.3389/fphys.2021.652896 (PMC8255669; doi:10.3389/fphys.2021.652896)
Supplement: Supplementary file 1 [file Table_1.docx]

Table 1. Mean bar velocity during the bench press exercise for six different loads.

| **Condition** | **40%1RM**  **(95%CI)** | **50%1RM**  **(95%CI)** | **60%1RM**  **(95%CI)** | **70%1RM**  **(95%CI)** | **80%1RM**  **(95%CI)** | **90%1RM**  **(95%CI)** |
| --- | --- | --- | --- | --- | --- | --- |
| **Peak Bar Velocity (m/s)** | | | | | | |
| **NO-BFR** | 1.04 ± 0.11  (0.96 to 1.11) | 0.94 ± 0.12  (0.86 to 1.03) | 0.83 ± 0.10  (0.76 to 0.90) | 0.73 ± 0.08  (0.68 to 0.79) | 0.65 ± 0.08  (0.59 to 0.70) | 0.53 ± 0.06  (0.49 to 0.57) |
| **I-BFR** | 1.01 ± 0.11  (0.94 to 1.09) | 0.92 ± 0.09  (0.86 to 0.98) | 0.83 ± 0.09  (0.77 to 0.89) | 0.75 ± 0.06  (0.71 to 0.80) | 0.66 ± 0.08  (0.60 to 0.71) | 0.57 ± 0.06  (0.53 to 0.61) |
| **C-BFR** | 1.00 ± 0.11  (0.93 to 1.08) | 0.93 ± 0.10  (0.87 to 0.99) | 0.83 ± 0.08  (0.78 to 0.89) | 0.73 ± 0.10  (0.66 to 0.79) | 0.61 ± 0.08  (0.55 to 0.67) | 0.53 ± 0.07  (0.48 to 0.58) |
| **Effect Size** | | | | | | |
| **NO-BFR vs I-BFR** | 0.27 | 0.19 | 0.0 | 0.28 | 0.13 | 0.67 |
| **NO-BFR vs C-BFR** | 0.36 | 0.09 | 0.0 | 0.0 | 0.50 | 0.0 |
| **I-BFR vs C-BFR** | 0.09 | 0.11 | 0.0 | 0.24 | 0.63 | 0.61 |

Results are expressed as mean ± SD (95% confidence intervals). Abbreviations: 1 RM=1 repetition maximum; NO-BRF= no blood flow restriction (control); I-BFR= intermittent blood flow restriction; C-BFR= continuous blood flow restriction.
