# Supplementary material for: Acute Effects of Different Blood Flow Restriction Protocols on Bar Velocity During the Squat Exercise
Source: Front Physiol. 2021 Jun 21;12:652896. doi: 10.3389/fphys.2021.652896 (PMC8255669; doi:10.3389/fphys.2021.652896)
Supplement: Supplementary file 2 [file Table_2.docx]

Table 2. Peak bar velocity during the bench press exercise for six different loads.

| **Condition** | **40%1RM**  **(95%CI)** | **50%1RM**  **(95%CI)** | **60%1RM**  **(95%CI)** | **70%1RM**  **(95%CI)** | **80%1RM**  **(95%CI)** | **90%1RM**  **(95%CI)** |
| --- | --- | --- | --- | --- | --- | --- |
| **Peak Bar Velocity (m/s)** | | | | | | |
| **NO-BFR** | 1.72 ± 0.24  (1.56 to 1.88) | 1.64 ± 0.27  (1.46 to 1.83) | 1.48 ± 0.19  (1.35 to 1.61) | 1.38 ± 0.21  (1.24 to 1.52) | 1.27 ± 0.18  (1.15 to 1.39) | 1.19 ± 0.15  (1.09 to 1.29) |
| **I-BFR** | 1.73 ± 0.25  (1.56 to 1.90) | 1.60 ± 0.21  (1.46 to 1.74) | 1.52 ± 0.18  (1.40 to 1.64) | 1.41 ± 0.13  (1.32 to 1.50) | 1.29 ± 0.13  (1.21 to 1.38) | 1.19 ± 0.10  (1.12 to 1.25) |
| **C-BFR** | 1.72 ± 0.24  (1.55 to 1.88) | 1.67 ± 0.19  (1.53 to 1.80) | 1.52 ± 0.15  (1.42 to 1.62) | 1.39 ± 0.15  (1.28 to 1.49) | 1.26 ± 0.16  (1.15 to 1.36) | 1.16 ± 0.14  (1.07 to 1.26) |
| **Effect Size** | | | | | | |
| **NO-BFR vs I-BFR** | 0.04 | 0.17 | 0.22 | 0.17 | 0.13 | 0.0 |
| **NO-BFR vs C-BFR** | 0.0 | 0.13 | 0.23 | 0.05 | 0.06 | 0.21 |
| **I-BFR vs C-BFR** | 0.04 | 0.35 | 0.0 | 0.14 | 0.21 | 0.25 |

Results are expressed as mean ± SD (95% confidence intervals). Abbreviations: 1 RM=1 repetition maximum; NO-BRF= no blood flow restriction (control); I-BFR= intermittent blood flow restriction; C-BFR= continuous blood flow restriction.
